# Development and Implementation of a Self-Optimizable Smart Lighting System Based on Learning Context in Classroom

**DOI:** 10.3390/ijerph17041217

**Published:** 2020-02-13

**Authors:** Baoshi Sun, Qiaoli Zhang, Shi Cao

**Affiliations:** 1 Department of Systems Design Engineering, University of Waterloo, Waterloo, ON N2L3G1, Canada; baoshi.sun@uwaterloo.ca; 2 Suzhou Shuyan Information Technology Ltd., 18F, 58 Qing Long Gang Rd, Suzhou 215000, China; zhangqiaoli1990@yeah.net

**Keywords:** smart classroom, smart lighting, learning context, LED lighting control, internet of things, environmental data-processing framework

## Abstract

Illumination is one of the most important environmental factors in the classroom. Researchers have discovered that lighting settings have significant impact on students’ performance. Although light-emitting diode (LED) lighting systems can precisely control brightness level and correlated color temperature (CCT), existing designs of LED lighting control systems for classrooms are focused on energy-saving but lack context-based illumination control ability. In this study, a smart lighting system with continuous evolution capability was developed. It can adjust brightness, CCT, and illuminance distribution dynamically according to specific learning context. This system allows not only manual control, but also automatic switching of scenes by integrating with school schedules. Based on existing knowledge about lighting preference, 10 lighting modes confined in the comfortable zone of Kruithof curve were proposed for various classroom scenarios. Moreover, a classroom environmental data-processing framework for collecting and analyzing learning context, illumination settings, environmental data, and students’ performance data was introduced. This framework can help researchers explore the correlation between student performance and environmental parameters.

## 1. Introduction

The classroom environment is an integrated indoor environmental system that consists of physical layout, interior design, infrastructure, furniture, and equipment, as well as indoor environmental factors including light, temperature, humidity, air quality, and acoustics. Existing studies have revealed that environment quality of classrooms has a significant impact on students’ wellbeing and performance [1,2,3,4,5,6,7]. Among all environmental factors, it is believed that light plays one of the most important roles. A study showed that light contributed 21% to the increase in student progress and marked the highest portion as compared to six other environmental factors [8].

Unfortunately, there is no simple solution for classroom lighting. Lang [9] argued that lighting preferences are not fixed but vary depending on many factors, such as classroom size, teaching activity, and individual needs of teachers and students. As a result, it is strongly advised that classroom lighting systems should provide users with the flexibility of lighting control. This opinion has been supported by data from empirical experiments. Studies demonstrated that different illumination configurations, including brightness and correlated color temperature (CCT) [10], had different impacts on students’ behavior and cognition, and the impact differed depending on learning context and age group [11,12,13,14,15,16,17,18]. On the other hand, with the development of LED products and Internet of Thing (IoT) technology, it is no longer a difficult task to perform precise and automatic control of brightness and CCT. Therefore, in a recent literature review, Sun el al. [19] concluded that the research focus in this field has shifted to the learning-context phase, with research and engineering priorities to discover and support better lighting configurations for different classroom activities.

Due to the variety of classroom contexts, the diversity of demographic characteristics of students, and the complexity of students’ response to different illumination settings, a sophisticated lighting control system is needed for the optimization of lighting environment for different conditions. Such a system, including both hardware and software, should meet the following requirements:

1. Can control both brightness level and CCT;
2. Can control based on zones in the classroom;
3. Allows automatic control of lighting based on classroom learning context, in additional to manual control;
4. Optimizes classroom lighting environment for best student performance by applying knowledge from scientific research;
5. Can take feedback such as students’ performance data and continuously improve lighting configurations.

In the current study, we designed and implemented an innovative smart lighting system in order to meet these requirements. The work is introduced in the following sections. Section 2 covers a literature review of lighting control systems; Section 3 describes the design and technical details of the proposed system; Section 4 proposes 10 lighting modes for different kind of learning contexts based on findings from existing studies; Section 5 presents a data-processing framework to collect and analyze environmental data and student performance data; Section 6 describes a case study of the proposed system; Section 7 summarizes this study and discusses future work.

## 2. Related Work

There have been many studies in the literature about lighting control systems. A comparison of them in terms of main purposes and techniques is shown in Table 1.

**Figure 1:**
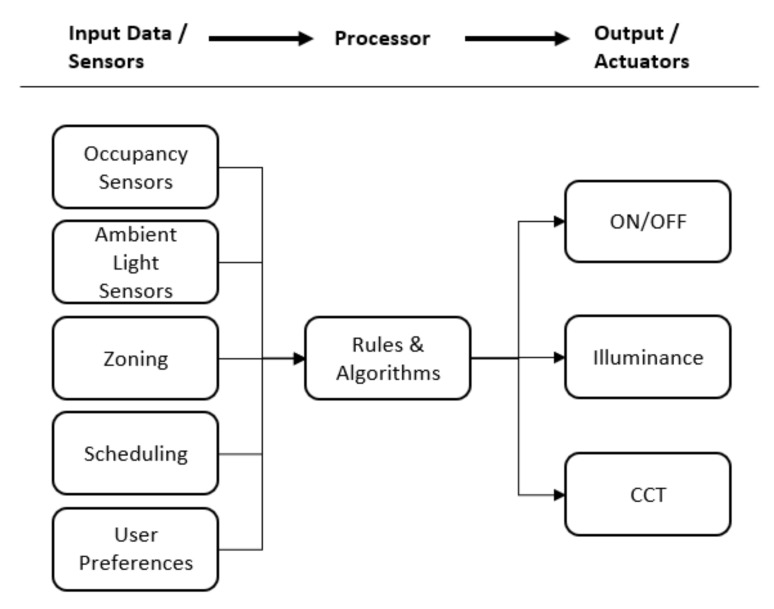
A general lighting control system model based on Martirano’s [20] control strategies.

As can be seen, the majority of studies on lighting control systems focused on energy saving [37], and they achieved considerable outcomes. As summarized in a review on lighting control technologies [38], the saving on energy ranged from 35% to 68% in classroom and office environment. Figure 1 depicts an abstracted lighting control system model for energy saving by referring to the control strategies suggested by Martirano [20]. Fundamentally, adopting high energy-efficiency LED and turning off or dimming the lights whenever possible are major methods to save energy. A study about the trend of lighting industry indicated that the benefits of promoting LED include not only energy saving and environment conservation, but also increasing automation [39]. Therefore, almost all pertinent studies used LED as the light source and applied on-off control. Most of them implemented brightness control at the same time, whereas a few of studies implemented CCT control. One motivation using CCT control to improve energy-efficiency might be the influence of different CCT on the occupant’s thermal perception. Some studies argued that occupants sensed warmer under lower CCT (warm light) environment [40,41], and this could lead to around 8% of the annual energy savings [41]. However, inconsistent reports can also be found, which rejected the hypothesis of correlation between CCT and thermal sensation [42]. Essentially, lighting control systems require relevant input data to decide when and how to control the lights. The input data employed by existing studies included different combinations of user configuration and sensor data, such as occupancy and ambient light, zone settings, schedule information, and user preference. Eventually, the core of the system is the algorithm that determines how to drive the lights according to the input data.

It is interesting to note that more and more researchers have focused on the improvement of control algorithms in recent years. Statistics, data modelling, and machine learning methods have been used in for energy saving purpose. For example, a neural network controller was designed and tested. It could control the lighting level of lamps in a classroom with regard to the ambient illuminance and the number of people [43]. In another study [44], in order to optimize the output of the lighting system by calculating daylight contribution, a data model based on statistics records was developed to determine the layout of lux sensors in large industrial buildings. Up to 80% energy saving on cloudy days was reported. Similarly, a statistical method was employed to optimize lighting control parameters, including sampling rate, converging speed, and error range of brightness, which achieved 55% or more energy savings [45]. In a later study [46], an advanced daylight harvesting model was proposed, which could map the daylight contribution from ceiling to workplaces. It was reported to have better energy efficiency comparing to a reference method.

Besides saving energy, some studies also considered to improve user experience [21,22,25]. Some researchers advocated the integrated lighting control with occupancy sensors, photocells, and central control module for users’ convenience and better experience [23]. A few researchers developed mobile applications for better user experience in terms of operability and mobility [18,30,31,36]. Evidence indicated that a well-designed mobile application can not only improve user experience, but also guarantee the compliance of illumination regulations and reduce energy consumption [36]. In other studies, both illuminance and CCT were considered for improving users’ visual comfort and wellbeing by adapting lighting environment to users’ activity [26,32].

It is noteworthy that more and more learning-context based lighting control systems have been recently proposed for classrooms [18]. These proposed systems followed existing psychological research findings by applying lower brightness and CCT for subjects like arts and language, and higher brightness and CCT for subjects like science and mathematics [31,32,33]. As summarized in Table 2, these systems provided illumination settings and control methods for a few basic learning scenarios. However, the lighting parameters and learning scenarios discussed in these studies were limited. First, the number of scenarios was not enough to cover the real-world educational activities. For example, elementary and secondary schools in China usually offer more than 10 subjects and activities; and the learning contents and teaching tools also keep changing with the development of economy and society. Second, the lighting configurations were either crude or static, without any dynamic and adaptive mechanisms. Third, the existing systems were only deployed in a few selected classrooms for experiments. Large-scale deployment for educational practice has not been reported.

In this study, we designed and implemented a learning-context-based smart lighting system, which provides more lighting scenarios and supports dynamic control of luminous level, CCT, and illuminance distribution. It has multiple objectives including students’ performance (most important), energy saving, manageability, and user experience. The system had been deployed in eight schools by the end of December 2019.

## 3. Learning-Context-Based Smart Lighting System

### 3.1. System Structure

**Figure 2:**
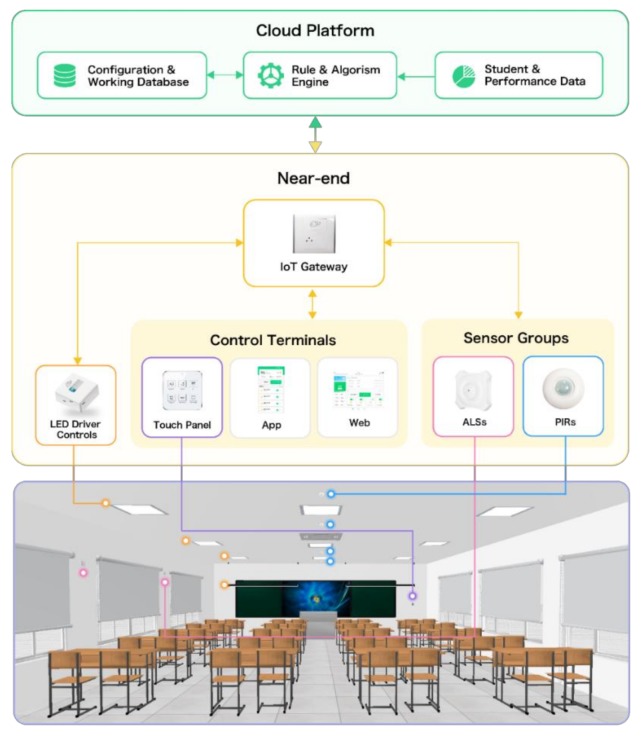
Structure of learning-context-based smart lighting system. ALS: Ambient light sensor; PIR: Passive infrared sensor.

As shown in Figure 2, the learning-context-based smart lighting system mainly consists of four parts at the near-end, including the IoT gateway, the LED lights with driver controls, the control panel, and the sensor groups, as well as a cloud platform at the far-end.

As the core device of the near-end system, IoT gateway, a.k.a. fog node, has a certain computational and storage capacity. It is the bridge between end-point devices and the cloud platform, responsible for message transfer and end-point devices management. One classroom is usually equipped with one IoT gateway.

In this design, each set of illumination unit includes an LED driver control and up-to-four LED fixtures. Each LED fixture supports dimming of brightness and CCT by means of PWM (Pulse Width Modulation). In order to save cost, one LED driver control is usually designated to one zone, where it requires isolated lighting control. For example, the classroom picture in Figure 2 delineates a typical configuration with four LED driver controls corresponding to four zones respectively, including one podium zone with two or three blackboard lights and three student zones (front, middle, and rear) with three classroom lights in each zone.

**Figure 3:**
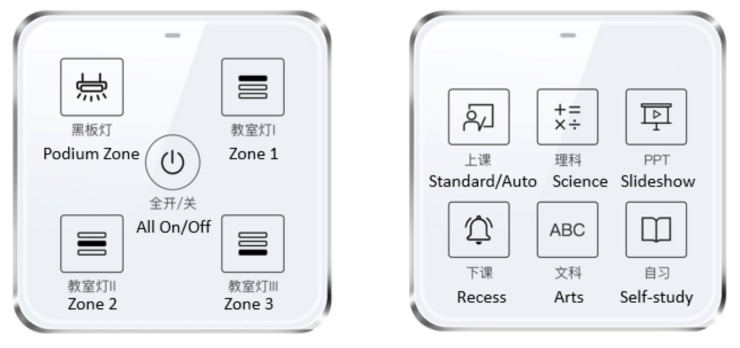
Control panels: Zone switch and scene control.

The control panel is a physical touch-panel with several function buttons. The control panel offers the user a convenient and quick way to operate the lighting system, including all lights on/off, zone on/off, and scene switching. Moreover, the button layout and function map of control panel can be customized according to users’ requirements. In this study, two types of control panel as shown in Figure 3 are designed to meet users’ preferences. The one with five touch-buttons is for zone-based switch. The other with six touch-buttons is for scene switching.

A sensor group is a combination of ambient light sensor (ALS) and/or passive infrared (PIR) sensor distributed in one classroom zone. The ALS is used to measure luminous level of the zone, and the PIR detects movement in the zone. By integrating information from multiple sensor groups, the system can determine the illuminance distribution and occupancy in a classroom.

The cloud platform communicates with IoT gateways via Wi-Fi. The cloud platform can remotely manage and control LED drive controls and other near-end modules through the IoT gateway in a classroom. Other system level functions, such as system configuration, system management, system monitoring, data storage, and analysis, are also provided by the cloud platform.

It is notable that the near-end devices in a classroom are designed as an autonomous local star network on RF2.4G (Radio Frequency 2.4GHz). The IoT gateway is the central node of the star topology, which has a replica of the classroom’s configuration. This means that the IoT gateway can independently manage the lighting system even if the connection to the cloud platform is lost. Such design ensures the robustness of the system. The loss of internet connection will only affect a few non-critical functions such as updating configuration, uploading sensor data, and internet remote control.

### 3.2. Hardware Design and Implementation

#### 3.2.1. IoT Gateway

**Figure 4:**
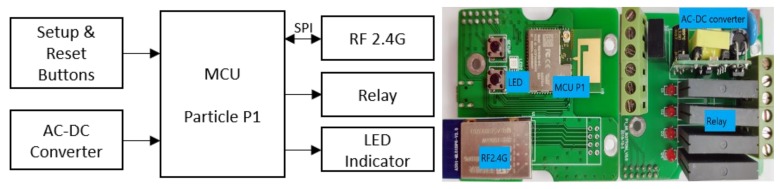
Internet of Things (IoT) gateway block diagram and working board.

Figure 4 shows the block diagram and the PCB (printed circuit board) of the IoT gateway. The main components include a 32-bit MCU (Microcontroller Unit) with built-in Wi-Fi, a RF2.4G module, a 4-way relay, and an AC to DC power module. Details can be found in Table 3. Each way of the relay controls the lighting circuit of one zone. Additionally, the gateway offers four GPIO (general-purpose input/output) ports, which can be used to connect external physical switches. This design provides a contingency plan. User can use the physical switches to turn on/off the lights of each zone, even when the local RF2.4G network is down.

#### 3.2.2. LED Driver Control

**Figure 5:**
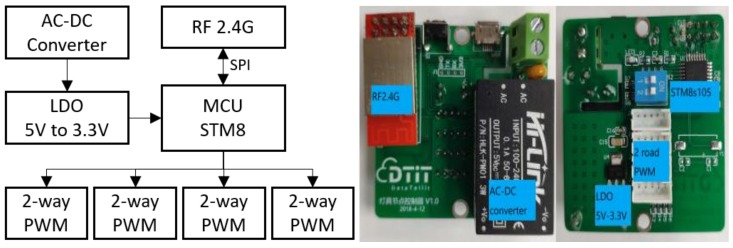
LED driver control block diagram and working board.

Figure 5 illustrates the block diagram and the PCB of the LED driver control, whose major parts, as listed in Table 4, include an 8-bit MCU, a RF2.4G module, and a set of power module. The LED driver control has four 2-way PWM ports. Each port can connect to a LED fixture’s power driver, and its 2-way PWM controls the cool white LED and the warm white LED, respectively, by controlling the duty ratio of the power output to adjust luminous level and CCT.

#### 3.2.3. Control Panel

**Figure 6:**
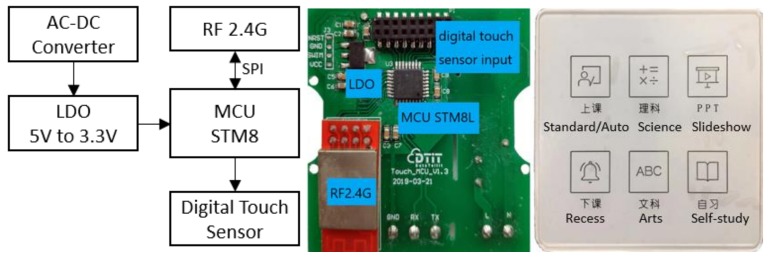
Control panel block diagram, working board, and front panel.

The block diagram and the PCB with its front panel of the control panel are demonstrated in Figure 6. The core component is an 8-key digital touch sensor, whose specification along with the main components are described in Table 5.

#### 3.2.4. Sensor Module

**Figure 7:**
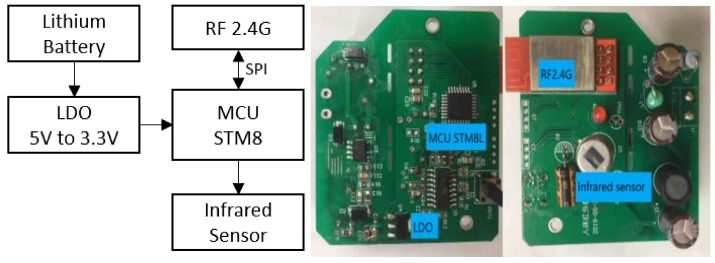
PIR sensor block diagram and working board.

In this study, passive infrared (PIR) sensors are utilized to detect the occupancy of classroom zones. Considering the size of zone, the detection distance of the chosen PIR sensor is about 3 to 5 m. Figure 7 shows the block diagram and the PCB of the PIR module, which mainly consists of an 8-bit MCU, a RF2.4G module, a power module, and a PIR sensor.

**Figure 8:**
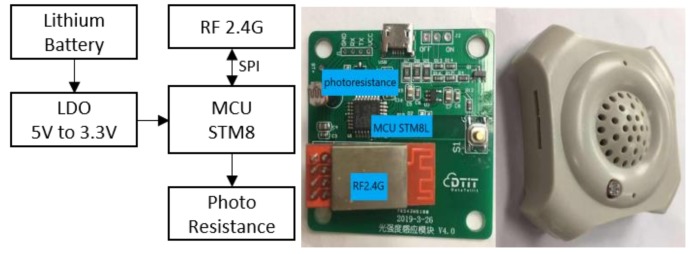
ALS block diagram, working board, and physical encapsulation.

The ambient light sensors (ALS) are employed to measure the luminous level in specific zones. Figure 8 displays the block diagram and the PCB of the ALS module, which mainly includes an 8-bit MCU, a RF2.4G module, a lithium battery, and a photo resistor. In this design, an ADC pin (analog to digital conversion) of the MCU is used for data sampling from the photo resistor. The module regularly works in low-power mode so that the battery life can reach about two years.

Table 6 describes the main components of PIR and ALS modules.

#### 3.2.5. LED Fixture and Power Driver

Two types of LED fixtures are utilized in this system, including the blackboard lights and the classroom lights. The size of blackboard lights is 1158 × 89.5 × 115.8 mm, and the classroom lights have two sizes: 595 × 595 × 10 mm and 1195 × 295 × 10 mm, which are applicable to ceiling and suspension installation, respectively. The LED fixture is equipped with both 3000 K (warm white) and 6500 K (cool white) LED beads, and its power driver has a 2-way PWM input port. By connecting the PWM input port to one of the PWM output ports of a LED driver control, the system can precisely control luminous level and CCT of the fixture. The specifications of the LED fixtures and the power driver are listed in Table 7.

### 3.3. Software Design and Implementation

#### 3.3.1. Embedded Software on IoT Gateway

**Figure 9:**
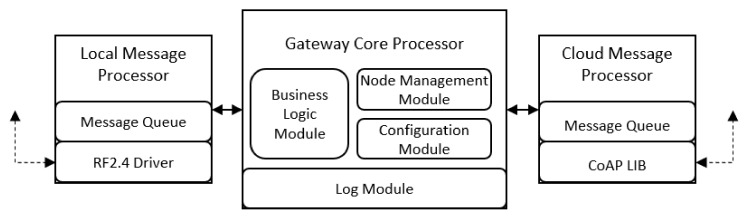
Software high-level design diagram of IoT gateway.

As shown in Figure 9, the embedded software on IoT gateway is composed of three major modules: One core processor and two message processors. The local message processor is responsible for message exchange within the local RF2.4G network, while the cloud message processor is in charge of communication with the cloud platform. The core processor primarily contains a node management module, a business logic module, a configuration module, and a log module. The node management module oversees registration, presence management, and real-time monitoring of sub-nodes at the near end. As the pivotal component of the core processor, the business logic module controls the procedure of business logic, such as scene switching, rule matching, and automation. The configuration module manages the settings of the gateway and synchronizes local configuration with the cloud platform if needed. The log module records the gateway’s working log, which can be used for system alerts and troubleshooting.

#### 3.3.2. Embedded Software on LED Driver Control

**Figure 10:**
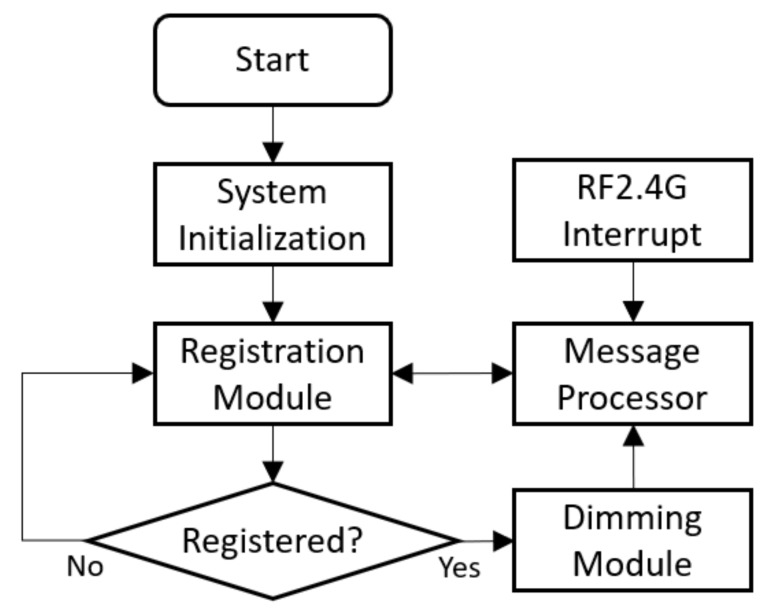
Software flowchart of LED driver control.

The embedded program running on STM8 MCU of the LED driver control basically includes registration module, message processor, and dimming module. As shown in Figure 10, to secure the communication, the LED driver control must register on the IoT gateway and get a token before any working message can go through. The message processor receives and parses control commands and reports the status of the LED fixture. The dimming module converts control commands into PWM control signals to manipulate luminous level and CCT of the LED fixture.

#### 3.3.3. Embedded Software on Control Panel

**Figure 11:**
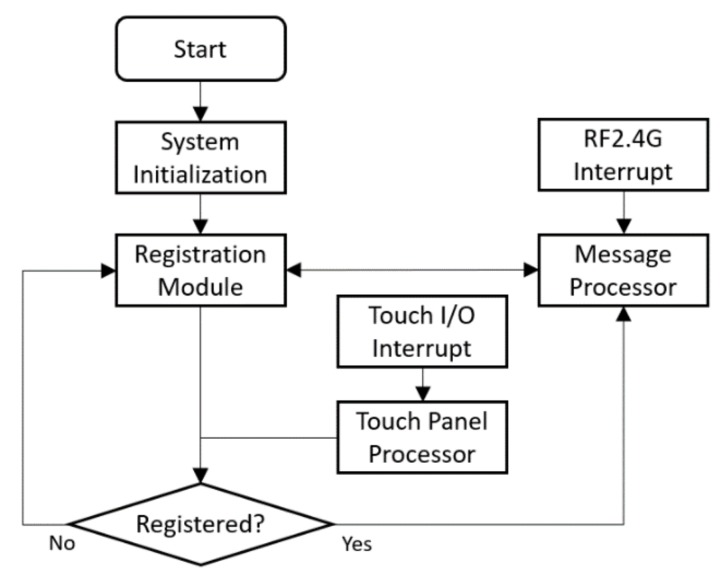
Software flowchart of control panel.

The control panel runs STM8 embedded program, which contains registration module, message processor, and touch panel processor. Figure 11 demonstrates the software workflow. The registration module and the message processor function the same as the modules of LED Driver Control. The touch panel processor takes key press signals and maps then to light control messages. Then, the control message is sent to the IoT gateway by the message processor.

#### 3.3.4. Embedded Software on Sensor Module

**Figure 12:**
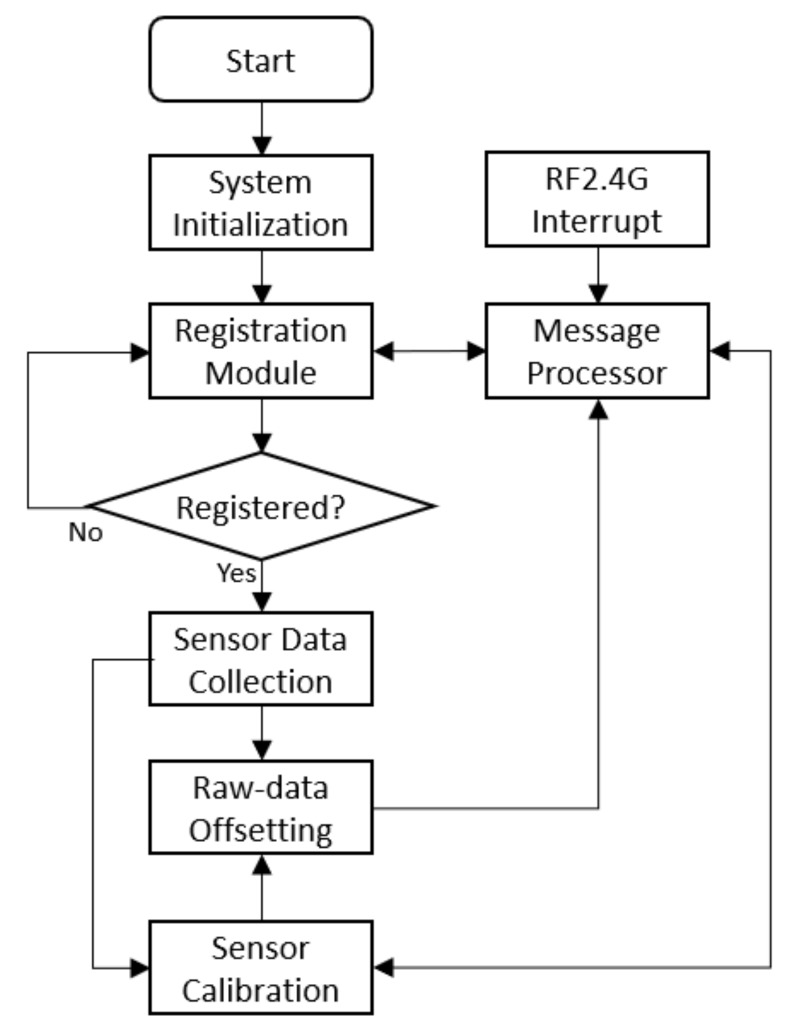
Software flowchart of sensor module.

The STM8 embedded program running on sensor module is comprised of registration module, message processor, sensor data collection module, raw-data offsetting module, and sensor calibration module. Figure 12 shows the software workflow. The registration module and the message processor work the same as those of LED driver control. The sensor data collection module gathers raw data from PIR or ALS sensor via digital or analog port of the MCU. ADC and TTL (transistor–transistor logic) are regular methods for raw-data sampling. Next, the raw data are processed by the raw-data offsetting module to compensate sensor drift. Then, the raw data are offset packed into RF2.4 messages and reported to the IoT gateway. To ensure the data consistency over time, the sensor calibration module reports the raw data to the cloud platform on specific occasion. For instance, the calibration of ALS is most likely at mid-night, although it may vary depending on location and outdoor environment. The algorithms on the cloud platform estimate the drift of sensors, determine the offset values, and feed back to the sensor calibration module. Details regarding these algorithms are not discussed in this article but will be discussed in a separate report.

#### 3.3.5. Cloud Platform

The cloud platform was an enhanced version of Spark-server, which is an open-source project on GitHub (https://github.com/particle-iot/spark-server). The major improvements performed by us focused on manageability and reliability, including system monitoring and alerting, gateway management, user and permission management, statistics, firmware version, and upgrade management.

#### 3.3.6. User Interface

**Figure 13:**
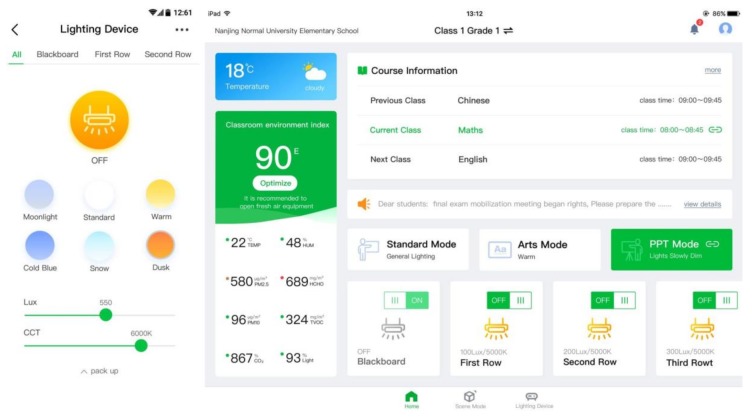
User interface of smart classroom lighting system (left: Smartphone; right: Tablet).

As demonstrated in Figure 13, the purposes of user interface (UI) of the suggested system can be roughly put into two categories: Control and monitoring. The control features of UI allow users to control each lighting fixture or each zone of lighting fixtures, switch lighting mode, as well as add user-defined mode and change the settings of existing lighting modes, including switch state, luminous level, and CCT. The monitoring features of UI give users intuitive views of real-time states and data for lighting fixtures and sensors. Additionally, all user interfaces of the system are built with HTML5 and suit both tablet and smartphone screen modes to meet the needs of different kinds of interaction scenarios.

### 3.4. Communication Interface

#### 3.4.1. Local Communication Protocol

In this study, the IoT devices in a classroom form a gateway-centered local star network on RF2.4G. Taking the advantages of RF2.4G, such as low power, small size, low cost, and easy configuration, the transmission distance of local network is confined within 20 m, which is sufficient for the communication within each classroom and avoids interference between adjacent classrooms.

In order to support both single-point and group control, each LED driver control in the near-end system will be assigned a node-ID and a zone mask. The range of node-ID is between 0 and 250, and ID 0 is reserved for the IoT gateway. A zone is represented by one bit in a byte, so the maximum number of zones in a classroom is eight. The zone mask is the sum of zones that the device belongs to. For example, the zone mask ‘00000101’ means zone 1 and zone 3.

A message between the IoT gateway and other endpoints is a data package, whose maximum size is 32 bytes. The first 7 bytes are the message head, and the rest are the message body, which varies from 0 to 25 bytes.

As shown in Table 8, the message head contains gateway ID, sender node-ID, receive node-ID, zone mask, message type, and command ID. There are three types of messages: Control message, query message, and internal message (e.g., configurations). Although a variety of commands are available, the light control commands and the sensor reporting commands are the most common ones. Moreover, the light control commands can control on/off, brightness, CCT, and special effects (e.g., breathing light), while the sensor reporting message can include just one data field or all eight data fields. Table 9; Table 10 describe the message body structure of these commands.

#### 3.4.2. Cloud Communication Protocol

The IoT gateway communicates with the cloud platform via Wi-Fi module, and the CoAP (Constrained Application Protocol) is employed for message transmission. The cloud messages are assembled into json format, and Table 11 gives some examples of them.

Furthermore, the service applications on the cloud platform speak to each other by means of message queue (Rabbit MQ), and https protocol is used for interaction between the UI and the backend services to achieve improved security.

### 3.5. Lighting Mode Control Flow

#### 3.5.1. Manual Switching

**Figure 14:**
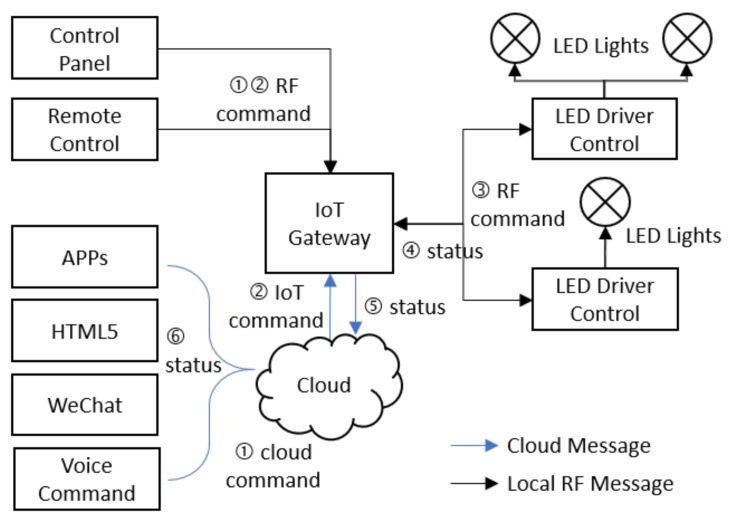
Flowchart of manual switching of lighting mode.

As shown in Figure 14, users have several ways to invoke a lighting mode switching instruction, such as control panel, remote control, software (e.g., APP, HTML5 page, WeChat) and voice command. The instruction is transmitted to the IoT gateway, then passed to the corresponding LED power controls by the gateway. On the other hand, the gateway gathers status of subordinate nodes and reports to the cloud. The cloud platform reflects the change of status on the UI.

#### 3.5.2. Class Schedule Linkage

**Figure 15:**
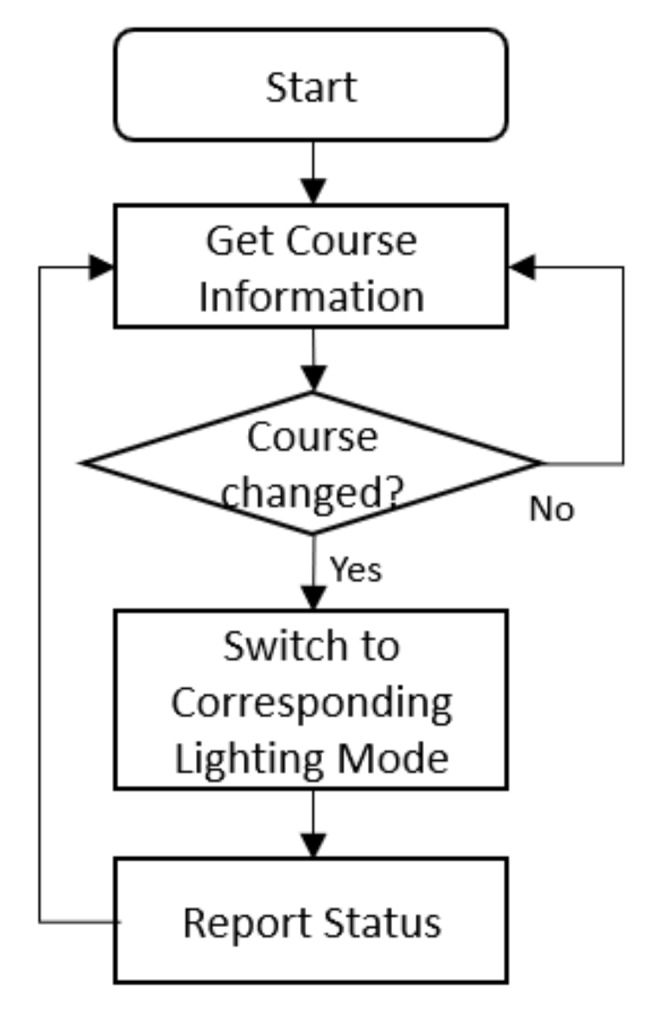
Flowchart of lighting mode switching with class schedules.

Besides manual operation, the suggested system also supports lighting mode and class schedule association. After the linkage is set, the system can automatically change lighting modes according to the current class context. The control flow is depicted in Figure 15.

### 3.6. Security and Reliability

To secure the communication between the IoT gateway and the cloud platform, DTLS (datagram transport layer security) is applied to the CoAP protocol. A gateway must possess a valid public key to register onto the cloud. At the cloud side, the frontend pages use https to call the backend services safely. At the near end, the token-based authentication mechanism is adopted to reinforce the security between the IoT gateway and the end points.

Considering that the 2.4G frequency band is widely shared by a lot of wireless devices, which may lead to high failure rate of communication due to signal interference, channel jamming, and message collision, in this study, we paid special attention to the reliability of the local RF2.4G network. Three techniques are applied for improving reliability, including time-divided transmitting, message queueing, and automatic resending. When multiple messages need to be sent at once, for example, to change the CCT of all LED fixtures to 5000 K, the gateway may generate four control messages (one for each LED driver control), and these messages are put into a queue with a sending timer. The sending timer of a message is calculated based on the node-ID of the destination. Because of the uniqueness of the node-ID, the values of sending timers will be properly spaced. In addition, any unsuccessfully transmitted message will be re-sent up to three times before being discarded. Moreover, continuous failures of message transmission will be reported to the cloud and trigger alarms if necessary.

Finally, the IoT gateway has the ability to function independently without the supervision of the cloud. This means that the IoT gateway can independently manage the lighting system even if the connection to the cloud platform is down.

## 4. Learning-Context-Based Lighting Modes

### 4.1. Ten Proposed Lighting Modes

In this study, we proposed 10 lighting modes to match different kinds of educational activities in classroom. Table 12 describes the settings and the purpose of each mode.

The proposed lighting modes were designed by consulting the conclusions from existing scientific studies in a comprehensive way, and complying with related lighting standards and regulations, like EN 12464-1:2011 (European Standard) [47], ANSI/IESNA RP-3-2006 (US Standard) [48], and (Chinese Standard) [49]. For example, brighter light (1000 Lux) was believed to improve vigilance and self-control capacity [13], and students showed more focused attention in challenging tasks under higher illuminance (1000 Lux) with higher CCT (6500 K) [11]. Meanwhile, students performed better for highly sensitive cognitive tasks [16] and 3D objects rotation tasks [50] under neutral white light (CCT = 4000 K), while variable light could reduce students’ restlessness and improve their social behaviors [15]. Furthermore, shifting CCT among 3500 K, 5000 K, and 6500 K was suggested in accordance with easy, standard, and intensive learning activities, respectively [18].

**Figure 16:**
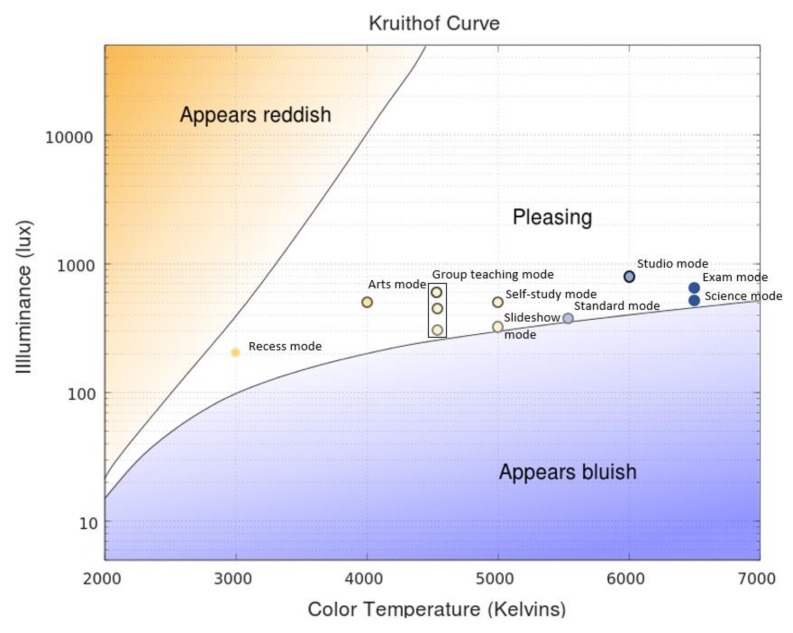
Proposed lighting modes within pleasing zone of Kruithof Curve.

The Kruithof curve [51] was employed to examine every proposed lighting mode. As shown in Figure 16, the combinations of illuminance and CCT of these lighting modes stay inside the pleasing zone.

### 4.2. Customization of Lighting Modes

**Figure 17:**
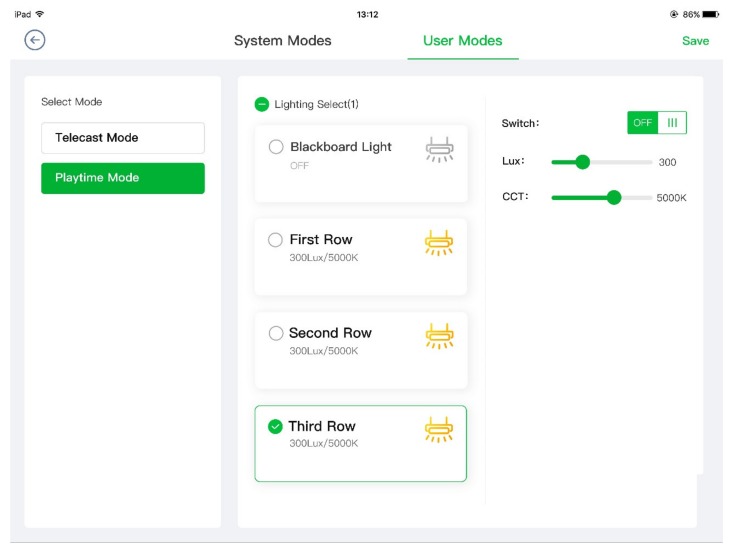
Tablet user interface (UI) of user-defined modes.

Since the basic lighting modes are fixed, they may not fit every situation well in practice. Given so many factors, such as the differences of physical layout and daylighting of classrooms, the variety of curriculums, the demographic diversity of students and the user preferences, which may require special lighting settings, there is a strong need for customized lighting modes. A UI for customization is implemented. As demonstrated in Figure 17, the user can modify existing modes and add new modes.

Furthermore, allowing user-defined settings is an effective way to accumulate data and learn from users. By continuously comparing the feedbacks or outcomes of different lighting settings for similar learning contexts, researchers will have the opportunity to optimize lighting configurations. There is still a lack of studies about learning-context based luminous environment. Existing studies have shown inconsistent results. For instance, some empirical results [52,53] did not support the suggestion that higher CCT benefits students in terms of focused attention and performance [11,18]. In the experiment of [11], contradictory results were found between participants from grade four and grade six. More data are needed to examine the effects of classroom lighting on student performance. To support large-scale data collection in future research and to provide optimal lighting for students, a classroom environmental data-processing framework is introduced, as described in the following section.

## 5. Classroom Lighting Environment Data-Collection and Self-Optimization Framework

Existing findings about lighting configuration and student performance were mostly obtained from controlled laboratory experiments. Such a method is limited due to small sample sizes, short test duration, and limited variables considered in each study. These restrictions were believed to be the major causes that led to inconsistency among existing results from previous studies [11,16,31]. Although the uses of IoT technology provide the context for new data sources, a real challenge is the integration of multiple systems and datasets [54]. A new approach to solve this problem is to utilize Big Data methodology and technology. By taking the advantage of Big Data and machine learning, in the current study. we proposed a classroom environmental data processing framework with the following features.

- Using student test results and exam grades to evaluate the impact of lighting configuration on students’ performance;
- Automatic data processing and parameter optimization;
- Allowing interaction and intervention of users;
- Generalizability and extensibility.

**Figure 18:**
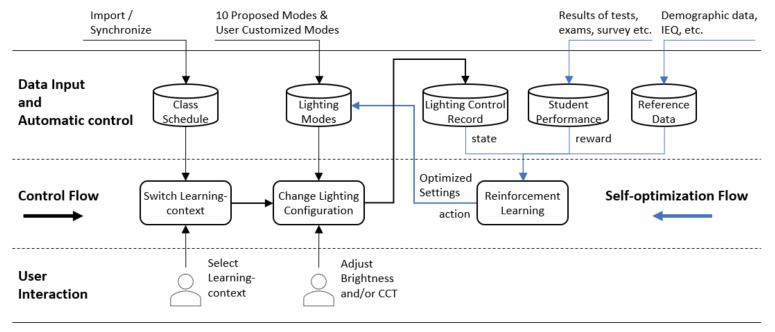
Classroom lighting environment self-optimization framework.

Figure 18 illustrates the mechanism and workflow of the proposed framework, which is comprised of two pivotal processes: The control flow and the self-optimization flow.

The control flow automatically reads class schedule and switches learning context accordingly, while the teacher can manually select learning context. Once learning context is changed, the system will load the corresponding lighting configuration from the lighting mode database, which initially contains the 10 proposed modes as introduced in Section 4.1. In this case, a user is allowed to customize lighting modes or apply ad-hoc settings on site. Any change of lighting system, whether automatic or manual, will be recorded for algorithm training.

The core part of the self-optimization flow is a set of learning agents of a reinforcement learning (RL) model. One agent corresponds to a specific learning context and its objective is to maximize the classroom’s average test score of the courses associated with that learning context. The agent takes the corresponding lighting configurations during a period (can be configured) as the current state of the environment. The agent uses the test scores of the corresponding courses during the same period to calculate the reward and determines how to optimize the lighting parameters for the learning context. Above is the basic idea of the RL model, whereas the detailed discussion of the algorithm is beyond the scope of this article and will be reported in a separate paper.

Theoretically, through long-term data accumulation and self-training, the lighting configuration can be gradually optimized. However, a few practical questions should be considered and dealt with.

- The demographic diversity of students should be considered. The system should support the input of demographic data, such as age and gender. Different learning agents of RL can be made for different demographic groups.
- The effects of other environmental factors need to be considered. The system should be able to collect other environmental data such as temperature, humidity, and CO_2_ density. Then, the scope of the RL can include more environmental variables in addition to lighting configuration in support of more comprehensive models.
- The changes of classrooms should be considered. Students usually change their classrooms in different grades. The system needs to record the changes and properly maintain the connection between students and their classroom assignment for the RL model.

Moreover, this framework can be extended by considering other dependent variables about student wellbeing such as myopia rate, sick leaves, and mental health. The data collected from this framework will support the analysis of the impact of classroom environmental factors on student wellbeing.

## 6. Case Study

**Figure 19:**
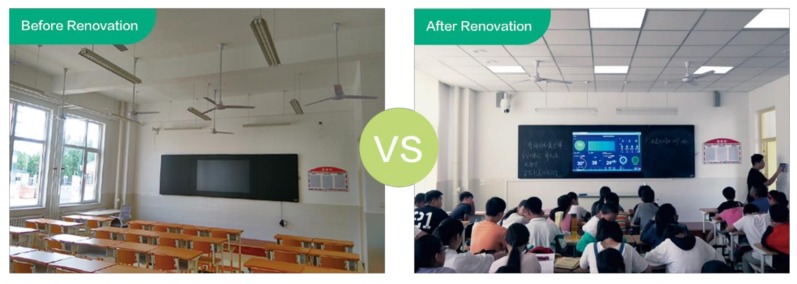
Comparison before and after classroom renovation.

The proposed smart lighting system has been deployed at several schools. One of them was deployed at an elementary school located in Hebei province, China in August 2017. In this project, the florescent tubes of 13 classrooms were replaced by the new LED smart lighting system. The pictures of before and after the replacement can be found in Figure 19.

Specifically, a classroom with smart lighting system has the following devices.

1. One IoT gateway,
2. Three ALSs to collect illuminance,
3. Four PIR sensors for occupancy detection on zone basis,
4. One integrated ambient environment sensor, which can sense a couple of environmental indices, including temperature, humidity, CO_2_ density, formaldehyde density, PM2.5 density, and PM10 density,
5. Two dimmable (both luminous level and CCT) LED blackboard-fixtures with the power driver,
6. Nine (3 by 3) dimmable (both luminous level and CCT) LED classroom-fixtures with the power driver. One row of three fixtures forms a zone,
7. One zone switch panel for turning zone lights on/off,
8. One scene control panel for fast switching lighting mode.

**Figure 20:**
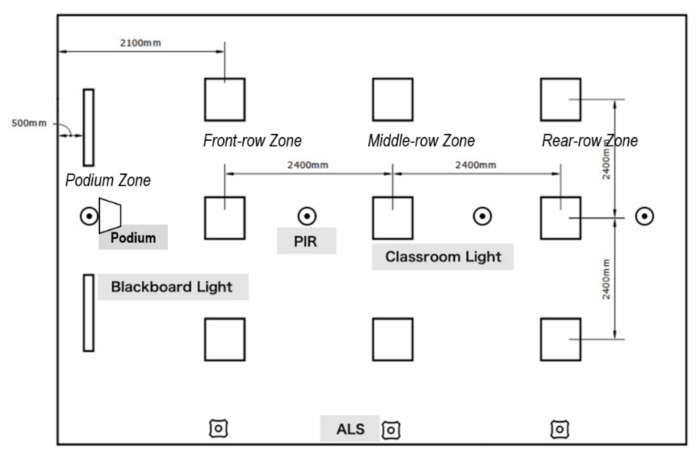
Standard classroom layout.

As shown in Figure 20, the classroom space is divided into four zones: The podium zone, the front-row zone, the middle-row zone, and the rear-row zone. The lights can be controlled individually, together, or by zone. Each zone is equipped with one PIR sensor to control the zone lights during non-working hours for energy saving. The three ALSs are distributed at the front, middle, and rear, respectively, on the side wall facing the windows.

**Figure 21:**
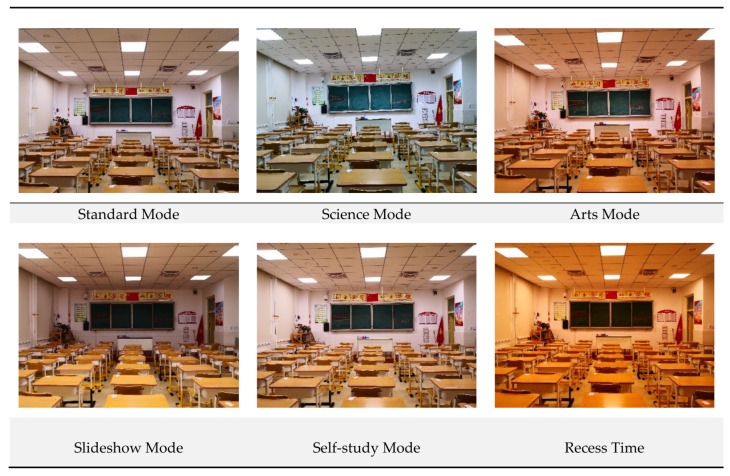
The six lighting modes applied in this case.

Upon the requirements of the school, six lighting modes were adopted, including the standard mode, the arts mode, the science mode, the slideshow mode, the self-study mode, and the recess time mode. Pictures in Figure 21 demonstrate the actual effect of these modes.

After the deployment, the user acceptance testing (UAT) was performed. The UAT results showed that the illuminance indicators of the lighting system, listed in Table 13, had reached or exceeded the national standards. Since then, data were continuously collected and sent to the cloud platform automatically for storage. Each classroom contributes about three mega-bytes of data per school day. The data include indoor environment indices, user operation log, as well as local outdoor environmental data, which were imported from open weather forecast data source.

In addition, the preliminary data of this deployment showed some interesting results. In general, teachers and parents who visited the classrooms were pleased with the smart lighting system. They also showed an interest in using such systems for offices and homes. User operation log indicated that older teachers preferred to use the wall panel to control the light and rarely used the mobile application or voice command. In contrast, the mobile application and voice commands were frequently used by younger teachers. Additionally, the smart features also induced students’ enthusiasm for new technology. As a result, the principal asked us about the possibility of developing some Do-It-Yourself workshops.

The accumulating data and positive feedbacks inspired us to do further investigation and analysis. A half-controlled field experiment involving 12 classrooms of grade one and grade two was launched in early September 2019 and will finish by June 2020. The 12 classrooms were equally divided into three groups. Group I included four classrooms without the smart lighting system. Group II and Group III were all equipped with the smart lighting system, but only Group III enabled the learning-context lighting modes, while Group II kept using only the standard mode with only manual on-off control. Students’ test results and exam grades were collected during the experiment. As this paper intends to introduce the smart lighting system from the technological perspective, the introduction of the experiment and detailed results, including the impact of the smart LED replacement, as well as the outcome of the application of learning-context-based lighting modes will be reported and discussed in another paper.

## 7. Conclusions and Future Study

By applying IoT and sensor technology, in this study, we designed and implemented a smart lighting system, which can dynamically control lighting environment in classrooms in accordance with the learning context. Unlike other research-oriented systems introduced in Section 2, the smart lighting system introduced in the current study not only supports research projects, but also satisfies large-scale deployment and day-to-day use. This is an advantage over existing commercial systems on the market, most of which are not smart or open enough. Existing proprietary solutions usually do not support customized development, nor give access to their database, which makes it difficult to integrate with other systems. Additionally, 10 lighting modes differentiated in luminous level, CCT, and light distribution were proposed to match the need of different learning contexts. Furthermore, a classroom environmental data-processing framework was introduced. The framework with RL algorithm model can perform self-optimization and explore better indoor environment settings by learning from environmental data and student performance data.

From the technological point of view, the system still has plenty of room for improvement. Some on-going tasks include integrating more kinds of devices, such as air conditioning, curtains, and fans into the system, supporting the self-calibration of sensors and the collaborative algorithm of multi-sensor, as well as improving the self-optimization framework and the RL model.

As a multi-objective design, the proposed smart lighting system primarily aims to improve students’ performance, with the consideration of energy saving, manageability, and user experience. Existing cases of implementing this system have demonstrated its feasibility. Teachers and school administrators can monitor classroom environment and set automated lighting schemes in a new way. The data collected from this system will help researchers study the impact of classroom environmental factors on student performance and wellbeing.

With the increasing deployment and continuous use of classroom smart lighting systems, more data will be accumulated to support researchers using Big Data tools to study the impact of classroom environment on students. This method has high validity and is better than traditional controlled laboratory experiments because it can collect a large amount of data from real classroom settings over a long period of time. The data will enable a wide range of future studies, for example human factors studies that analyze users’ preferences when multiple control methods are provided, vision health studies that investigate the effect of classroom lighting conditions on student myopia occurrence, and public health studies that explore the relationship between classroom environmental factors and student mental health and wellbeing, as well as the interactions between environmental variables. The results from such studies will help examine current classroom lighting guidelines and standards and suggest improvements for the benefits of students. The smart lighting system introduced in this study provides a technical foundation for future studies.

## Figures and Tables

**Table 1:** Comparison of studies on lighting control systems.

Research	Main Purposes				Techniques and Methods							
	Energy Saving	Manageability	Better User Experience	Students’ Performance Improvement	Occupancy Detection	Ambient Light Sensing	Control by Zones	Scheduling	Dimming (Adjustment of Luminous Level)	Adjustment of Color Temperature	Mobile Application	Learning Context Awareness
**Martirano, L. [20]**	**√**		**√**		**√**	**√**	**√**	**√**	**√**			
**J. Byun et al. [21]**	**√**		**√**		**√**	**√**	**√**		**√**			
**May and Yaseen. [22]**	**√**		**√**		**√**	**√**	**√**		**√**			
**Middleton et al. [23]**	**√**		**√**		**√**	**√**	**√**		**√**			
**Martirano, L. [24]**	**√**				**√**	**√**	**√**		**√**			
**Parise et al. [25]**	**√**		**√**		**√**	**√**	**√**	**√**	**√**			
**Kwon, S.-Y. et al. [26]**	**√**		**√**		**√**		**√**	**√**	**√**	**√**		**√**
**Li, M. et al. [27]**	**√**	**√**				**√**			**√**			
**M. Rossi et al. [28]**	**√**				**√**	**√**			**√**			
**Kamienski et al. [29]**	**√**				**√**	**√**			**√**			
**Suresh et al. [30]**	**√**		**√**		**√**		**√**				**√**	
**Choi and Suk. [18]**				**√**						**√**	**√**	**√**
**Moon et al. [31]**			**√**	**√**		**√**		**√**	**√**	**√**	**√**	**√**
**Lee et al. [32]**	**√**		**√**	**√**	**√**			**√**	**√**	**√**		**√**
**Zhong et al. [33]**	**√**		**√**	**√**	**√**				**√**	**√**		**√**
**Zhang and Liu. [34]**	**√**				**√**	**√**	**√**					
**T. de Rubeis et al. [35]**	**√**				**√**	**√**			**√**			
**Castillo-Martinez et al. [36]**	**√**	**√**	**√**	**√**		**√**	**√**		**√**		**√**	
**This Study**	**√**	**√**	**√**	**√**	**√**	**√**	**√**	**√**	**√**	**√**	**√**	**√**

**Table 2:** Learning scenarios and lighting parameters of reference studies.

Study	No. of Learning Scenarios	Subject	Illumination Settings	
			CCT (K)	Brightness (Lux)
Moon et al. [31]	5	Concentration/Mathematics	6500	600
		Language/Society	4500	400
		Creativity/Arts	3500	300
		Rest	3500	180
		Sleep	3000	<10
Lee et al. [32]	4	Mathematics and Science	5000–6000	600
		Language/Memorizing	4000–5000	400
		Arts	3000–4000	300
		Rest	N/A	N/A
Zhong et al. [33]	6 ^1^	Arts, Science, Recess, Rest, Self-study and Exam	3000–5500	>300

^1^ Focused on the functions and technical implementation but did not specify corresponding settings for each scenario.

**Table 3:** Main components of IoT gateway.

Type	Component Model	Picture	Description
**MCU**	Particle P1	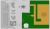	STM32 120 Mhz ARM Cortex M3
			Broadcom BCM43362 Wi-Fi chip
			1 M flash, 128 K RAM
**RF2.4G**	nRF24L01P	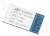	SPI
			Distance 20 m
			Power 20 dBm 100 mw
**Relay**	G3MB-202P	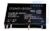	4-way
			electric current 2A
**Power**	NJ02-AXXL	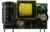	AC220 V to DC5 V
			400 mA

**Table 4:** Main components of LED driver control.

Type	Component Model	Picture	Description
**MCU**	STM8S105K4	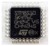	8 bit
			32 K flash
			2 K RAM
**RF2.4G**	nRF24L01P	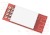	SPI
			Distance 10 m
			Power 0 dBm 1 mw
**Power**	HLK-PM01	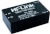	AC100 ~ 240 V to DC5 V/3 W
**LDO**	AMS1117	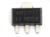	5–3.3 V
			0 ~ 1 A

**Table 5:** Main components of control panel.

Type	Component Model	Picture	Description
**MCU**	STM8L151K4	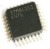	8 bit
			32 K flash
			2 K RAM
**RF2.4G**	nRF24L01P	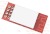	SPI
			Distance 10 m
			Power 0 dBm 1 mw
**Power**	HLK-PM01	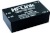	AC100 ~ 240 V to DC5 V/3 W
**LDO**	AMS1117	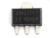	5–3.3 V
			0 ~ 1 A
**Digital Touch Sensor**	TTP226	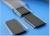	8 touch keys
			2 ~ 5.5 V
			−20 °C to +70 °C

**Table 6:** Main components of sensor modules.

Type	Component Model	Picture	Description
**MCU**	STM8L151K4	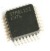	8 bit
			32 K flash
			2 K RAM
**RF2.4G**	nRF24L01P	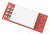	SPI
			Distance 10 m
			Power 0 dBm 1 mw
**Battery**	Lithium Battery	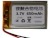	3.7 V/650 mAH
**LDO**	TPS782	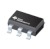	5 V–3.3 V
			0 ~ 1 A

**Infrared Sensor**	RE200B	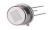	Angle 120, Distance 3 ~ 5 m
			3 ~ 10 V, −30 °C to +70 °C
**Photo Resistor**	5516	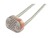	10 Lux 5 ~ 10 KΩ
			Max Power 9 mW, 0 ~ 150 V
			−30 °C to +70 °C

**Table 7:** LED lighting fixtures and power driver.

Class	Size	Description	Picture
**Blackboard Light**	Size: 1158 × 89.5 × 115.8 mm	LM 2700 LM	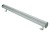
	Installation: Pendant	LX 1200 Lux	
		CCT 3000 K ~ 6500 K	
**Classroom Light**	Size: 595 × 595 × 10 mm	LM 3050 LM ~ 3550 LM	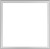
	Installation: Ceiling	LX ≥ 500 Lux	
		CCT 3000 K ~ 6500 K	
**Classroom Light**	Size: 1195 × 295 × 10 mm	LM 3050 LM ~ 3550 LM	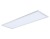
	Installation: Pendant	LX ≥ 500 Lux	
		CCT 3000 K ~ 6500 K	
**Power Driver**	Size: 155 × 53 × 30 mm	Input 110 ~ 240 VAC	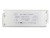
		Output 33 ~ 40 Vdc	
		2-way PWM Input	
		950 mA × 2	
		Power 36 W	
		PF > 0.95s	

**Table 8:** Message head of RF2.4G message.

Gateway	Sender	Receiver	Version & Length	Message Type	Command	Zone Mask
8 bit	8 bit	8 bit	8 bit	8 bit	8 bit	8 bit

**Table 9:** Message body structure for light control command.

On/Off	On/Off	Bright	On/Off	Bright	CCT	On/Off	Effects
8 bits	8 bits	8 bits	8 bits	8 bits	16 bits	8 bits	8 bits

**Table 10:** Message body structure for sensor reporting command.

ALS	PIR	Temp.	Humidity	PM25	CO_2_	TVOC	CH_2_O
8 bits	8 bits	16 bits	16 bits	16 bits	16 bits	16 bits	16 bits

**Table 11:** Cloud message examples.

Function	Example	Description
Set Switch	{‘cmd’:1, ‘nd’:1, ‘state’:1}	Turn #1 light on
	{‘cmd’:1, ‘nd’:1, ‘state’:0}	Turn #1 light off
Set Brightness	{‘cmd’:3, ‘nd’:1, ‘value’:60}	Set #1 light to 60% brightness
Set CCT	{‘cmd’:5, ‘nd’:1, ‘value’:3500}	Set CCT of #1 light to 3500K
Set Light State	{‘cmd’:2, ‘nd’:1, ‘ring’: (0,1,60,3500)}	Turn on #1 light with 60% brightness and 3500 K CCT

**Table 12:** Ten proposed lighting modes for common learning contexts.

Learning Context	Illumination Settings		Purposes
	Teacher Zone	Student Zone	
* Standard	350 Lux, 5500 K	350 Lux, 5500 K	Regular settings can act as default configuration
* Science	800 Lux, 6500 K	500 Lux, 6500 K	Enhance alertness and arousal, better for science courses like physics, math, chemistry, etc.
* Arts	800 Lux, 5000 K	500 Lux, 4000 K	Bright and warm environment can inspire creativity, better for courses like music, painting, language, etc.
* Recess Time	Off	200 Lux, 3000 K	Easy and relaxing lights for better rest and recovery for next class
* Slideshow	Off	5000 K, Front/Middle/Rear: 100/200/300 Lux	Better screen vision for slideshow
* Self-study	Off	500 Lux, 5000 K	More focus on one’s own work
* Class Over	Off	Off	Energy saving
Exam	Off	650 Lux, 6500 K	Altering and concentrating yields better results
Performing	800 Lux, 6000 K	Off	Center of attention
Group Teaching	450 Lux, 4500K	On team working, keep overhead lights of each team at 450 Lux and 4500 K; On presenting of specific team, dim up the lights of that team to 600 Lux and dim down those of other teams to 300 Lux.	Encourage interaction within team members, but suppress disturbance between teams

Note: those with “*” mark are frequently used lighting modes, which can be activated on the scene control panel. The recess-time mode and the class-over mode share the ‘Recess’ button. The exact action will be determined by the occasion when the button is pressed. During the school time, the recess-time mode will be triggered. Otherwise, the class-over mode will be triggered.

**Table 13:** The checked illuminance indicators during the project user acceptance testing (UAT).

Indicator	National Standard ^1^	UAT Results ^2^
Illuminance Level (desktop)	≥300 Lux	435 Lux
Illuminance Uniformity (desktop)	≥0.7	0.81
Illuminance Level (blackboard)	≥500 Lux	589 Lux
Illuminance Uniformity (blackboard)	≥0.8	0.85
Unified Glare Rating (UGR)	<19	15.6
Color-rending Index (CRI)	≥80	95

^1^ GB 7793-2010. Hygienic Standard for Day Lighting and Artificial Lighting for Middle and Elementary School [49]; ^2^ Tested under the standard mode.

## References

[1] Earthman G.I. (2004). Prioritization of 31 Criteria for School Building Adequacy.

[2] Higgins S., Hall E., Wall K., Woolner P., McCaughey C. (2005). The Impact of School Environments: A Literature Review.

[3] Blackmore J., Bateman D., Loughlin J., O’Mara J., Aranda G. (2011). Research into Learning Spaces and between Built the Connection Student Outcomes.

[4] Yang Z., Becerik-Gerber B., Mino L. (2013). A study on student perceptions of higher education classrooms: Impact of classroom attributes on student satisfaction and performance. Build. Environ..

[5] Gilavand A., Jamshidnezhad A. (2016). Investigating the Impact of Educational Spaces Painted on Learning and Educational Achievement of Elementary Students in Ahvaz, Southwest of Iran. Int. J. Pediatr..

[6] Zhang Y., Barrett P., Davies F., Barrett L. (2016). A field survey on the indoor environmental quality of the UK primary school classroom. Br. J. Sch. Nurs..

[7] Ministry of Education (2016). Gabrielle Wall The Impact of Physical Design on Student Outcomes.

[8] Barrett P., Davies F., Zhang Y., Barrett L. (2015). The impact of classroom design on pupils’ learning: Final results of a holistic, multi-level analysis. Build. Environ..

[9] Lang D. (2002). Teacher Interactions within the Physical Environment: How Teachers Alter Their Space and/or Routines Because of Classroom Character.

[10] Borbély Á., Sámson Á., Schanda J. (2001). The concept of correlated colour temperature revisited. Color Res. Appl..

[11] Sleegers P., Moolenaar N., Galetzka M., Pruyn A., Sarroukh B., van der Zande B. (2013). Lighting affects students’ concentration positively: Findings from three Dutch studies. Light. Res. Technol..

[12] Singh P., Arora R. Classroom Illuminance: Its impact on Students’ Health Exposure & Concentration Performance. Proceedings of the International Ergonomics Conference HWWE 2014.

[13] Smolders K.C.H.J., de Kort Y.A.W. (2014). Bright light and mental fatigue: Effects on alertness, vitality, performance and physiological arousal. J. Environ. Psychol..

[14] Keis O., Helbig H., Streb J., Hille K. (2014). Influence of blue-enriched classroom lighting on students’ cognitive performance. Trends Neurosci. Educ..

[15] Wessolowski N., Koenig H., Schulte-Markwort M., Barkmann C. (2014). The effect of variable light on the fidgetiness and social behavior of pupils in school. J. Environ. Psychol..

[16] Fabio B., Chiara B., Ornella L.R., Laura B., Simonetta F. (2015). Non Visual Effects of Light: An Overview and an Italian Experience. Energy Procedia.

[17] Ballina M. (2016). Illuminating Education: Composition and Use of Lighting in Public K-12 Classrooms. Ph.D. Thesis.

[18] Choi K., Suk H.-J. (2016). Dynamic lighting system for the learning environment: Performance of elementary students. Opt. Express.

[19] Sun B., Li Z., Cao S. (2018). The Impact of Classroom Lighting on Student Performance: A Literature Review. Chin. J. Appl. Psychol..

[20] Martirano L. (2011). A smart lighting control to save energy. Proceedings of the 6th IEEE International Conference on Intelligent Data Acquisition and Advanced Computing Systems.

[21] Byun J., Hong I., Lee B., Park S. (2013). Intelligent household LED lighting system considering energy efficiency and user satisfaction. IEEE Trans. Consum. Electron..

[22] May Z.B., Mohd Yaseen Y.A.A.B. (2013). Smart Energy Saving Classroom System Using Programmable Logic Controller. Adv. Mater. Res..

[23] Middleton-White S., Smith G., Martin R., Hartnagel T.J., Weber T.E., Crane M., Arbouw T., Kack D.R., Rector D.J. (2013). Integrated Lighting System and Method. U.S. Patent.

[24] Martirano L. (2014). A sample case of an advanced lighting system in a educational building. Proceedings of the 2014 14th International Conference on Environment and Electrical Engineering.

[25] Parise G., Martirano L., Cecchini G. (2014). Design and Energetic Analysis of an Advanced Control Upgrading Existing Lighting Systems. IEEE Trans. Ind. Appl..

[26] Kwon S.-Y., Im K.-M., Lim J.-H. (2014). LED Context Lighting System in Residential Areas. Sci. World J..

[27] Li M., Lu S.L., Wu R.R., Wang G.W. (2015). Design and Implementation of Classroom Intelligent LED Lighting Control System. Appl. Mech. Mater..

[28] Rossi M., Pandharipande A., Caicedo D., Schenato L., Cenedese A. (2015). Personal lighting control with occupancy and daylight adaptation. Energy Build..

[29] Kamienski C., Borelli F., Biondi G., Rosa W., Pinheiro I., Zyrianoff I., Sadok D., Pramudianto F. (2015). Context-aware energy efficiency management for smart buildings. Proceedings of the 2015 IEEE 2nd World Forum on Internet of Things (WF-IoT).

[30] Suresh S., Anusha H.N.S., Rajath T., Soundarya P., Vudatha S.V.P. (2016). Automatic lighting and Control System For Classroom. Proceedings of the 2016 International Conference on ICT in Business Industry & Government (ICTBIG).

[31] Moon S.-M., Kwon S.-Y., Lim J.-H. (2016). Implementation of smartphone-based color temperature and wavelength control LED lighting system. Cluster Comput..

[32] Lee H.-S., Kwon S.-Y., Lim J.-H. (2016). A Development of a Lighting Control System Based on Context-Awareness for the Improvement of Learning Efficiency in Classroom. Wirel. Pers. Commun..

[33] Zhong X., Hou H., Qiao Q. (2016). Application of LED Intelligent Lighting in the Classroom of the Primary and Secondary School. China Illum. Eng. J..

[34] Zhang X.-Z., Liu L.-S. (2018). Design of the Classroom Intelligent Light Control System Based on ARM9. International Conference on Smart Vehicular Technology, Transportation, Communication and Applications.

[35] De Rubeis T., Muttillo M., Pantoli L., Nardi I., Leone I., Stornelli V., Ambrosini D. (2017). A first approach to universal daylight and occupancy control system for any lamps: Simulated case in an academic classroom. Energy Build..

[36] Castillo-Martinez A., Medina-Merodio J.-A., Gutierrez-Martinez J.-M., Aguado-Delgado J., De-Pablos-Heredero C., Otón S. (2018). Evaluation and Improvement of Lighting Efficiency in Working Spaces. Sustainability.

[37] Chew I., Karunatilaka D., Tan C.P., Kalavally V. (2017). Smart lighting: The way forward? Reviewing the past to shape the future. Energy Build..

[38] Haq M.A.U., Hassan M.Y., Abdullah H., Rahman H.A., Abdullah M.P., Hussin F., Said D.M. (2014). A review on lighting control technologies in commercial buildings, their performance and affecting factors. Renew. Sustain. Energy Rev..

[39] Li Z., Jia P., Zhao F., Kang Y. (2018). The Development Path of the Lighting Industry in Mainland China: Execution of Energy Conservation and Management on Mercury Emission. Int. J. Environ. Res. Public Health.

[40] Golasi I., Salata F., de Lieto Vollaro E., Peña-García A. (2019). Influence of lighting colour temperature on indoor thermal perception: A strategy to save energy from the HVAC installations. Energy Build..

[41] Toftum J., Thorseth A., Markvart J., Logadóttir Á. (2018). Occupant response to different correlated colour temperatures of white LED lighting. Build. Environ..

[42] Baniya R.R., Tetri E., Virtanen J., Halonen L. (2018). The effect of correlated colour temperature of lighting on thermal sensation and thermal comfort in a simulated indoor workplace. Indoor Built Environ..

[43] Chen Y., Sun Q. (2013). Artificial intelligent control for indoor lighting basing on person number in classroom. Proceedings of the 2013 9th Asian Control Conference (ASCC).

[44] Wang L., Li H., Zou X., Shen X. (2015). Lighting system design based on a sensor network for energy savings in large industrial buildings. Energy Build..

[45] Chew I., Kalavally V., Oo N.W., Parkkinen J. (2016). Design of an energy-saving controller for an intelligent LED lighting system. Energy Build..

[46] Borile S., Pandharipande A., Caicedo D., Schenato L., Cenedese A. (2017). A Data-Driven Daylight Estimation Approach to Lighting Control. IEEE Access.

[47] European Committee for Standardization EN 12464-1:2011 (2011). Light and Lighting. Lighting of Work Places. Part 1: Indoor Work Places.

[48] Illuminating Engineering Society ANSI/IES RP-3-13 (2014). American National Standard Practice on Lighting for Educational Facilities.

[49] GB 7793-2010 (2011). Hygienic Standard for Day Lighting and Artificial Lighting for Middle and Elementary School.

[50] Ferlazzo F., Piccardi L., Burattini C., Barbalace M., Giannini A.M., Bisegna F. (2014). Effects of new light sources on task switching and mental rotation performance. J. Environ. Psychol..

[51] Davis R.G., Ginthner D.N. (1990). Correlated Color Temperature, Illuminance Level, and the Kruithof Curve. J. Illum. Eng. Soc..

[52] Park Y. (2015). Color temperature’s impact on task performance and brainwaves of school-age children. J. Phys. Ther. Sci..

[53] Smolders K.C.H.J., de Kort Y.A.W. (2017). Investigating daytime effects of correlated colour temperature on experiences, performance, and arousal. J. Environ. Psychol..

[54] Bublitz F.M., Oetomo A., Sahu K.S., Kuang A., Fadrique L.X., Velmovitsky P.E., Nobrega R.M., Morita P.P. (2019). Disruptive Technologies for Environment and Health Research: An Overview of Artificial Intelligence, Blockchain, and Internet of Things. Int. J. Environ. Res. Public Health.

